# *detectMITE*: A novel approach to detect miniature inverted repeat transposable elements in genomes

**DOI:** 10.1038/srep19688

**Published:** 2016-01-22

**Authors:** Congting Ye, Guoli Ji, Chun Liang

**Affiliations:** 1 Department of Automation, Xiamen University, Xiamen, Fujian 361005, China; 2 Department of Biology, Miami University, Oxford, Ohio 45056, USA; 3 Innovation Center for Cell Biology, Xiamen University, Xiamen, Fujian 361102, China

## Abstract

Miniature inverted repeat transposable elements (MITEs) are prevalent in eukaryotic genomes, including plants and animals. Classified as a type of non-autonomous DNA transposable elements, they play important roles in genome organization and evolution. Comprehensive and accurate genome-wide detection of MITEs in various eukaryotic genomes can improve our understanding of their origins, transposition processes, regulatory mechanisms, and biological relevance with regard to gene structures, expression, and regulation. In this paper, we present a new MATLAB-based program called *detectMITE* that employs a novel numeric calculation algorithm to replace conventional string matching algorithms in MITE detection, adopts the Lempel-Ziv complexity algorithm to filter out MITE candidates with low complexity, and utilizes the powerful clustering program *CD-HIT* to cluster similar MITEs into MITE families. Using the rice genome as test data, we found that *detectMITE* can more accurately, comprehensively, and efficiently detect MITEs on a genome-wide scale than other popular MITE detection tools. Through comparison with the potential MITEs annotated in Repbase, the widely used eukaryotic repeat database, *detectMITE* has been shown to find known and novel MITEs with a complete structure and full-length copies in the genome. *detectMITE* is an open source tool (https://sourceforge.net/projects/detectmite).

Transposable elements (TEs, also called mobile elements) are a type of repeat sequences abundant in eukaryotic genomes^1^,^2^,^3^,^4^. TEs play important roles in genome organization and evolution^5^,^6^. Commonly, TEs in genomes can be classified into two major categories – retrotransposons (Class I) and DNA transposons (Class II). Movement of retrotransposons utilizes a “copy-and-paste” strategy requiring RNA intermediates, while DNA transposons transit through a “cut-and-paste” mechanism without RNA intermediates^7^,^8^,^9^. Miniature inverted repeat transposable elements (MITEs) are a special type of DNA transposons, which share the common feature of DNA transposons, *i.e.*, containing short conserved terminal inverted repeats (TIRs), but have higher copy numbers in genomes like retrotransposons^10^,^11^. As shown in Fig. 1, a typical MITE is composed of an internal sequence and a TIR pair (≥10 *nt* in length). The TIR pair flanks the internal sequence, and the whole MITE is then flanked by a direct repeat pair called a target site duplication (TSD, 2–10 *nt* in length). MITEs vary in length from 50 to 800 *nt*. Generally, MITEs do not encode proteins and have no coding potential for their transposition, and therefore are non-autonomous TEs. However, they frequently locate in introns in genic regions or close to gene ends in intergenic regions^12^,^13^. Considering that genes are often associated with MITEs, a PCR-based genome mapping and fingerprinting technology called Inter-MITE Polymorphism (IMP) was developed to take advantage of MITE-based genomic markers^14^. Because of the polymorphism in the insertion or transposition sites of the MITE *mPing* in different rice cultivars, MITEs were used to generate molecular markers to perform quantitative trait loci (QTL) analysis between these closely related genomes^15^.

MITEs are abundantly distributed in eukaryotic genomes, including plants and animals. Their movements in genomes can change the structures and functions of genes. In the potato, a MITE named *stowaway* was found to cause phenotypic variation of tuber skin color through its insertion into the first exon of flavonoid 3′,5′-hydroxylase gene^16^. Genome-wide MITE analysis in rice has shown that MITEs contribute to genome diversity, novel gene emergence and mRNA transcript variations^17^. In *Oryza sativa*, genes distant from MITEs were found to have higher expression than those adjacent to MITEs or containing MITEs^13^. Comparative analysis of MITEs in *Brassica rapa*, *Brassica oleracea*, and *Arabidopsis thaliana* demonstrated that MITEs play dynamic roles in genome evolution of the *Brassica*^18^.

Comprehensive and accurate detection of MITEs on a genome-wide scale can facilitate our understanding of their origins, transposition mechanisms, and regulatory roles in genome organization and gene structure, expression, and regulation^17^,^19^,^20^. With rapid improvements in sequencing technologies and drops in sequencing costs, more and more genomes from various species are available for studying MITEs. The major bioinformatics methods in TE identification can be classified into three groups: *de novo*, structure-based, and homology-based methods^21^,^22^. *de novo* methods focus on the innate characteristic of TEs (*i.e.*, repetition) to discover hidden TEs in genomes, without any prior information (*e.g.* structure or sequence similarities with known TEs). *de novo* methods are suitable for identifying both known and novel TEs, but detection results often contain a mixture of different types of TEs and non-TE repeats, which necessitate further classification and filtration. Structure-based methods identify subsequences of the defined structures of known TEs in genomes. They can detect special type of TEs, but have the drawback of identifying many TEs with low copy members and/or non-TE repeats in their detection outputs. Using programs like BLAST^23^, RepeatMasker^24^ and HUMMER3^25^, homology-based methods utilize sequence similarities between putative and known TEs to detect TEs hidden in genomes. They are good at detecting real TEs, even those with a single copy in genomes. However, they cannot detect novel TEs, and have detection results that frequently contain sequences without a full-length copy or complete structure of well-defined TEs.

Based on the well-defined structures of MITEs and sequence similarity among different MITE homologs, several computational tools have been developed to detect MITEs in DNA sequences. As a structure-based method, *FINDMITE*^26^ was designed to detect MITEs in the African malaria mosquito (*Anopheles gambiae*). It requires users to predefine the TSD sequences, TIR length, and the minimum and maximum distances between the TIRs. All putative MITE sequences meeting these requirements will be retained, except TIRs with high A/T or C/G content or TIRs including simple repeats^26^. Another structure-based method, MITE Uncovering SysTem (*MUST*)^27^ uses a string matching algorithm to detect sequences with a TIR pair within a window ≤500 *nt* and retains those sequences flanked by TSDs. After retrieving all putative MITE candidates, *MUST* groups them into MITE families based on the sequence similarity of the internal sequences between TIR pairs^27^. Unfortunately, both *FINDMITE* and *MUST* were demonstrated to have high false positive rates in MITE detection and cannot deal with genome-scale inputs^21^.

Considering that different members of a MITE family have different flanking sequences and using multiple sequence alignment to identify MITE members, *MITE-Hunter* has successfully decreased the false positive rate in MITE detection^21^. Assuming MITEs are randomly distributed in genomes, *MITE Digger*^28^ is able to detect MITEs in full genomes using a computational strategy that processes a smaller portion of genome at a time. *MITE Digger* has shown a significant improvement in detection efficiency, as demonstrated for the rice genome (*i.e.*, ~15 hours). Both *MITE-Hunter* and *MITE Digger* utilized a mixture of both *de novo* and structure-based methods in MITE detection. Although they have successfully decreased false positive rates in MITE detection, both *MITE-Hunter* and *MITE Digger* cannot detect all MITEs hidden in the genomes^29^. As a *de novo* method, the program *RSPB* (Repetitive Sequence with Precise Boundaries) also used a string matching algorithm to discover the repetitive sequences in genomes that have precise boundaries^13^. Compared with *MITE-Hunter* and *MITE-Digger*, *RSPB* can find more MITEs, but its output often contains lots of sequences that bear short/diverse TIRs (*i.e.*, TIR pairs with a lower degree of pairing) or have no TSD. Moreover, many sequences present as a single copy in the output of *RSPB* are unlikely to be a real MITE.

Several databases (*e.g.*, Repbase^30^,^31^, P-MITE^29^, BrassicaTED^32^) provide MITE annotations for different species. As the most widely used database of eukaryotic repetitive and transposable elements, Repbase^30^,^31^ contains different types of repeat elements, including MITEs, from various species. P-MITE^29^ is a database for MITEs detected in 41 plant species using *MITE-Hunter*, *MITE Digger* and *RSPB*. BrassicaTED is a specialized database for *Brassica* species, which contains MITEs, TRIMs (Terminal Repeat Retrotransposon in Miniatures), and SINEs (Short Interspersed Elements).

Generally speaking, there are three main challenges in genome-wide detection of MITEs: (1) the rapid, comprehensive and accurate detection of putative MITE sequences in genomes, (2) the effective filtration of false positive cases from putative MITE candidates, and (3) the efficient clustering of similar MITE sequences into distinctive MITE families. To address these challenges, we developed a novel MATLAB-based program called *detectMITE*, which employs a complex-number-based numeric calculation to replace conventional string matching algorithms in MITE detection on a genome scale. To filter out false positives, we adopted the Lempel-Ziv complexity algorithm for filtering low-complexity sequences and utilized a filtration strategy that is based on sequence similarity among MITE flanks^21^. *detectMITE* uses an effective and accurate clustering program called *CD-HIT*^33^,^34^ to cluster similar MITEs into distinctive MITE families. Our comparative data analysis shows that *detectMITE* can more comprehensively, accurately, and efficiently detect MITEs on a genome-wide scale than *MITE-Hunter*, *MITE Digger* and *RSPB*, all of which are capable of processing genome-scale inputs.

## Methods

**Figure 1:**
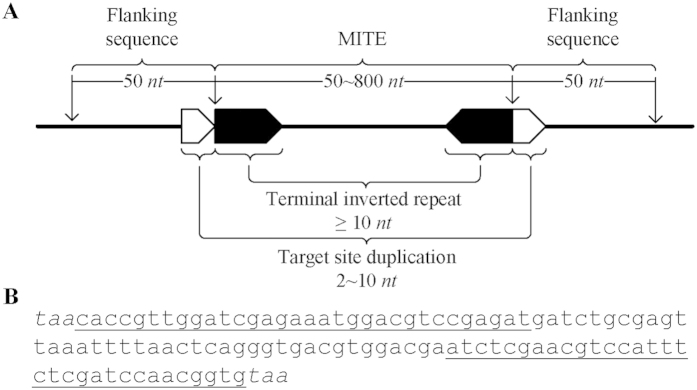
The typical structure of miniature inverted repeat transposable element (MITE). (**A**) A complete structure of MITE, not including target site duplication (TSD). (**B**) An example sequence of MITE flanked by TSD. The underlined bases represent a terminal inverted repeat (TIR) pair while the bases in italics represent a direct repeat pair (TSD).

**Figure 2:**
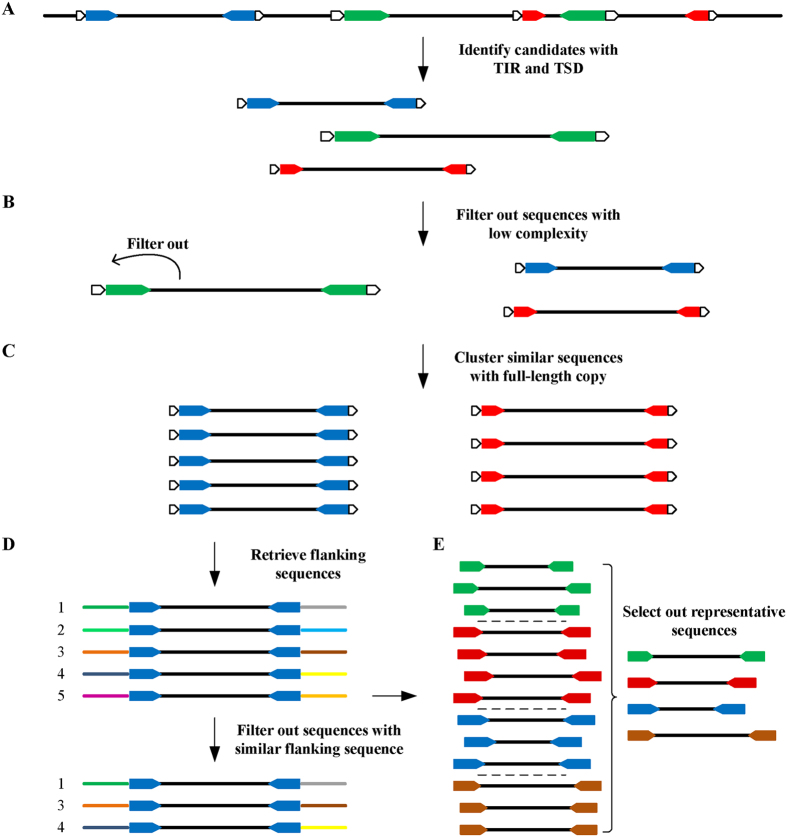
The core algorithm and flow chart of *detectMITE* in MITE detection. (**A**) Detection of MITE candidate sequences with TIR and TSD. (**B**) Filtration of MITE candidates with low complexity. (**C**) Clustering of similar MITEs into MITE families. (**D**) Filtration of MITE family members in terms of their flanking sequence similarity. (**E**) Selection of the representative sequence for each MITE family with ≥3 valid members that have different flanking sequences.

Replacing conventional string matching algorithms for inverted repeat detection, we have created *findIR*^35^, which utilizes prime-number-based numeric calculation and manipulation to identify perfect inverted repeats, and *detectIR*^36^, which deploys complex-number-based numeric calculation for detecting both perfect and imperfect inverted repeats. Both tools have demonstrated their capability to more efficiently, accurately, and comprehensively detect perfect and imperfect inverted repeats than other popular tools^35^,^36^. As non-autonomous DNA transposons, MITEs are characterized by their terminal inverted repeats. Consequently, the core algorithm of *detectIR* in inverted repeat detection has been adopted and modified by *detectMITE*. Due to special structure requirements of MITEs (as shown in Fig. 1) and other constraints, *detectMITE* required new functions, including detection of target site duplication, clustering of similar MITE candidates into distinctive MITE families, and reducing false positive cases of MITEs. As shown in Fig. 2, the core algorithm of *detectMITE* includes the following five main steps:

### Detection of MITE candidate sequences with TIR and TSD

For a given genome, all sequence fragments that contain a TIR pair at their ends (default length = 10 *nt*, see Fig. 1), being flanked by a TSD (2–10 *nt*), and have a length between 50 and 800 *nt* will be identified in this step. First, each genomic sequence input (*i.e.*, individual chromosome sequences) will be mapped into a numeric vector of complex numbers using the mapping score schema: (A → 1, T → −1, C → j, G → −j). As the score summation of the subsequence’s corresponding vector, the cumulative scores will be calculated for all subsequences with a length of 10 *nt*. If the sum of the cumulative scores of any two subsequences located within a range of 50~800 *nt* is ***C*** (a complex number), and if the sum of the absolute values of the real part and the imaginary part of ***C*** is ≤2, then the two subsequences are potential terminal inverted repeats. Next, the potential TSD - a direct repeat pair flanking the TIR pair - will be searched and validated (*i.e.*, the cumulative scores for the two target sites must be exactly the same, and the two target sites have the same length of 2–10 *nt*). Through robust numerical vector calculation of MATLAB, all subsequences, *i.e.*, MITE candidate sequences with the same length, can be searched exhaustively and validated efficiently. Since numerical calculation enables an efficient and exhaustive search, all putative MITEs that meet the defined criteria will be identified and kept for the downstream analysis. *detectIR*^36^ can detect both perfect inverted repeats with two completely reverse complementary halves (stem) and imperfect inverted repeats with a middle non-palindromic spacer (loop) and non-complementary pairs in the stem. Unfortunately, in its most recent version it cannot detect inverted repeats with indels inside the stem^36^. Correspondingly, *detectMITE* is also incapable of detecting MITEs with indel(s) in their terminal inverted repeats. Even with this limitation, *detectMITE* has demonstrated its capability for more accurate and comprehensive detection of MITEs on a genome scale in comparison with three popular tools (see **Results**).

### Filtration of MITE candidates with low complexity

Because low complexity sequences are rare in real MITEs^21^, we need to filter out MITE candidates having low complexity in their sequences. The DUST program^37^ integrating BLAST has been often used to identify low complexity sequences^38^,^39^,^40^. This program has also been utilized by *MITE-Hunter*^21^ to filter out MITE candidates with low complexity. In *detectMITE*, we replaced DUST with the Lempel-Ziv complexity algorithm, which is frequently used in biosignal analysis^41^,^42^. As shown in Supplementary Fig. S1, our Lempel-Ziv complexity analysis for MITEs identified by *MITE-Hunter* and *RSPB* indicated that many reported MITEs still have low complexity sequences, which are unlikely to be valid MITEs. In *detectMITE*, each putative MITE that meets one of the following criteria was filtered out as a false positive: (1) the TIR contains a homopolymer or dinucleotide stretch of a length ≥8 *nt*, (2) the TIR contains low G/C or A/T content (default <20%), (3) the Lempel-Ziv complexity value of the sequence is less than 0.675, and (4) if the target site length is 2, the target site is not ‘TA’. Similar criteria have been adopted by others to reduce false positive cases of MITEs^21^,^26^,^28^.

### Clustering of similar MITEs into MITE families

As transposable elements, MITEs move within genomes, leading to multiple copies distributed along the whole genomes. Accordingly, filtering out putative MITEs with low mobility (*i.e.*, low copy number) in genomes can effectively reduce the false positive cases in MITE detection. The prerequisite for determining and counting the copy number of a specific putative MITE candidate is to cluster identical or highly similar candidate MITEs with full copy lengths together. Among the existing tools for genome-wide MITE detection (*e.g.*, *MITE-Hunter*^21^, *MITE Digger*^28^, and *RSPB*^13^), blastn-based clustering approaches have been utilized to cluster similar MITEs into MITE families. Because blastn-based clustering is usually time-consuming and reports fragmented sequences^33^, *CD-HIT* was adopted in *detectMITE*. *CD-HIT* adopts short word filters and a greedy strategy to avoid unnecessary comparisons and reduce redundant computations dramatically in clustering^33^,^34^,^43^. In *detectMITE*, candidate MITEs with similarity (*i.e.*, the number of match bases/the length of the shorter sequence) ≥80% and coverage rate (*i.e.*, the aligned length/the length of the longer sequence) ≥99% will be grouped into the same MITE families. After clustering, MITE families containing few members will be filtered out (*i.e.*, having fewer than 3 members).

### Filtration of MITE family members in terms of their flanking sequence similarity

When a MITE is transposed into different genomic locations, it is less likely that its flanking sequences will also be transmitted together^21^,^28^. Therefore, within a given MITE family generated from the aforementioned clustering step, we will keep the valid MITE members that have different flanking sequences in order to count the copy number of this family across the entire genome conservatively, reducing false positives. To compare flanking sequences, we extracted 50 *nt* sequences from both sides of a candidate MITE (see Fig. 1), and conducted pairwise alignments to identify sequence similarity. For a given MITE family, left flanks and right flanks are compared respectively using pairwise alignments; no comparison is conducted between left and right flanks. If two left (or right) flanks share at least 50% similarity (*i.e.*, ≥25 bases matched in their pairwise alignments), only one MITE will be kept in this MITE family. Finally, all valid members retained for a given MITE family must have different left and right flanking sequences. As shown in Fig. 2D, a MITE family has 5 full-length copies (putative MITE candidates), left flanking sequences of candidate 1 and candidate 2 have high similarity, and the right flanking sequences of candidate 4 and candidate 5 have high similarity. Candidate 2 and candidate 5 were removed in this step, so the family has 3 full-length valid members that have different left and right flanking sequences.

### Selection of the representative sequence for each MITE family with enough members

After the filtration process in the previous step, the MITE families with at least 3 valid members were retained as valid families, while others were recognized as invalid, false positive cases and filtered out. For each valid MITE family, we will select a representative sequence to represent that family (see Fig. 2E). If a family has *n* distinctive valid members, the similarity score (*i.e.*, optimal local alignment score) between any two member sequences *i* and *j* is *score(i, j).* The *representativeness_score* of sequence *i* is defined as,


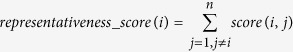


Here, sequences mean the valid MITE members flanked by a TSD pair. Then, the sequence with the highest score will be selected as the representative sequence. *MITE-Hunter* uses multiple sequence alignment of each family to generate a consensus sequence to represent the corresponding family. In *detectMITE*, we use representative sequences to replace consensus sequences that may contain mismatches/indels due to multiple sequence alignment, ensuring that the representative sequences can be unambiguously positioned in the genome.

Unlike *MITE-Hunter*, *MITE Digger* and *RSPB*, we use strict criteria when clustering similar MITEs into MITE families. For instance, in *MITE-Hunter*, a MITE is validated if it has at least three full-length copies characterized by TIRs and flanking TSDs, and all MITE sequences are clustered into MITE families using the 80-80-80 rule from all-against-all blastn results^21^: *i.e.*, two sequences will be classified into the same family if both of them have a length of ≥80 *nt* and share sequence similarity of ≥80% in at least 80% aligned sequences. In contrast, *RSPB* adopts the E-value of ≤10^−10^ rule to generate final MITE families^13^: *i.e.*, two sequences will be classified into the same family if they have a valid blastn hit with an E-value of ≤10^−10^. Apparently, loose clustering criteria in *MITE-Hunter*, *MITE Digger* and *RSPB* tend to cluster similar MITE sequences into a smaller number of MITE families with more members whereas strict clustering criteria in *detectMITE* would result in a larger number of smaller MITE families. The rationale for us to do this is to retain the completeness and validity of MITE members within a given MITE family as best we can, without losing accurate structural information that can be advantageous in further downstream data analyses including genome annotation. For a given MITE family generated using loose clustering criteria, a representative or consensus sequence cannot always represent faithfully the sequence and structural characteristics of all MITE members within that family. In contrast, the representative sequence of a MITE family generated using strict clustering criteria can be directly used to retrieve its members in the genome with precise boundaries and high sequence similarity.

The entire algorithms were implemented into a package of MATLAB scripts, which require pre-installation of *CD-HIT*^33^,^34^. *detectMITE* is an open-source tool (https://sourceforge.net/projects/detectmite). All the tests were performed using Ubuntu 12.04 (precise) 64-bit platform with Intel Xeon (2.00 GHz) processors, 4 CPU cores and 128 GB RAM.

## Results

To test the performance of *detectMITE*, we used *detectMITE* to detect MITEs in the *Oryza sativa* genome (MSU Rice Genome Annotation Project Release 6.1) and compared the detection results using *MITE-Hunter*, *MITE Digger*, and *RSPB* (see Table 1; outputs of each tool are available at: http://sourceforge.net/projects/detectmite/files/Supplementary_Data.7z).

As shown in Table 1, *detectMITE* took 10.79 hours to detect 35969 MITE sequences that have a complete structure with TIR and TSD in the rice genome, which were clustered into 4790 MITE families. In contrast, *MITE-Hunter* took 28.01 hours to detect 631 MITE families, each of which has a consensus sequence generated from multiple sequence alignment. Among these 631 consensus sequences, 578 have a length between 50 and 800 *nt*. *MITE Digger* took 15.44 hours to identify 332 MITE families, each of which has a representative sequence^28^. *RSPB* identified 179415 MITE sequences using more time than *MITE Digger*, and used blastn (E-value ≤10^−10^) to group them into 497 families^29^,^13^. Obviously, *detectMITE* is more efficient than these popular tools. Apparently, *RSPB* identified many more MITEs than the other three tools, but the majority of its detected MITEs (*i.e.*, 68.6%) lack the complete structure of a typical MITE.

To evaluate MITE detection accuracy, the detection result of *detectMITE* was compared with both Repbase^30^ and the outputs of *MITE-Hunter*, *MITE Digger*, and *RSPB* individually. Since P-MITE database contains a mixture of outputs from *MITE-Hunter*, *MITE Digger*, and *RSPB*^29^, P-MITE is not used for our comparison. Because *detectMITE*, *MITE-Hunter*, and *MITE Digger* only detect MITEs with complete structures, we filtered out all partial MITE sequences in the output of *RSPB* and kept 56391 sequences that were labelled as complete sequences for comparison (see Table 1).

### Comparison of the outputs of *MITE-Hunter*, *MITE Digger*, *RSPB* and *detectMITE* with Repbase data

**Figure 3:**
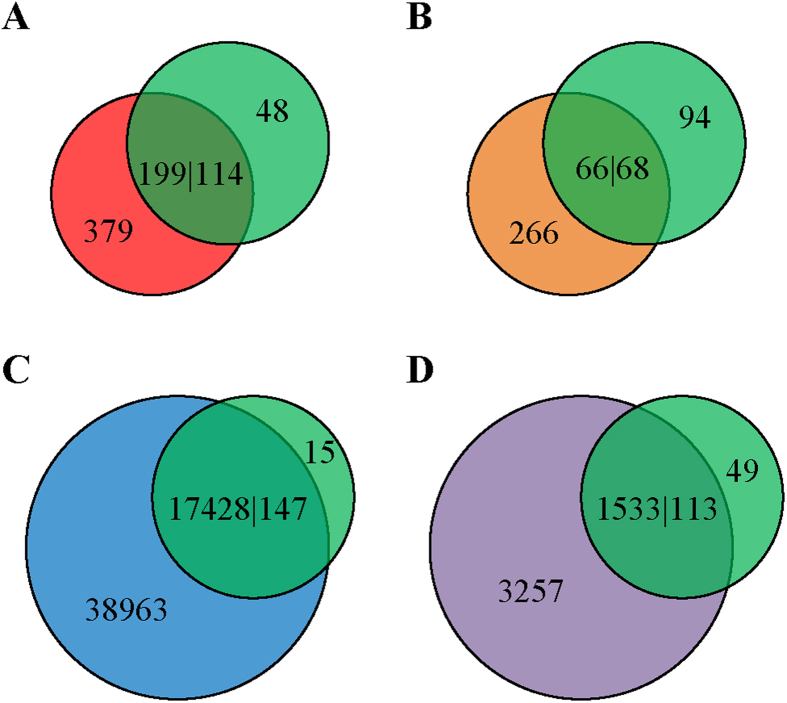
The comparisons of MITEs detected in the rice genome between Repbase reference data and the outputs of *MITE-Hunter*, *MITE Digger*, *RSPB* and *detectMITE* respectively. (**A**) Comparison of *MITE-Hunter* outputs with the Repbase data. (**B**) Comparison of *MITE Digger* outputs with the Repbase data. (**C**) Comparison of *RSPB* outputs with the Repbase data. (**D**) Comparison of *detectMITE* outputs with the Repbase data. The green circle represents the Repbase reference data whereas red, brown, blue and violet circles represent the outputs of *MITE-Hunter*, *MITE Digger*, *RSPB* and *detectMITE* respectively. The overlapping parts represent numbers of MITE sequences that match each other by blastn (E-value ≤10^−10^). Using the *detectMITE* result (**D**) as an example, in the right-bottom graph, 1533|113 means 1533 sequences of *detectMITE* output match 113 sequences of Repbase reference data, 3257 represents the number of MITE sequences in *detectMITE* output that do not match any sequences in the Repbase data, and 49 represents the number of MITE sequences in the Repbase data that do not match any sequences in the output of *detectMITE*.

Repbase is a comprehensive repeat database that contains both transposon elements and other repeats such as tandem repeats^30^,^44^. It has been widely utilized in genome annotation^45^,^46^,^47^. As short non-autonomous DNA transposons (Class II), MITEs are not explicitly annotated and labeled in the current Repbase release^30^,^31^. Therefore, we extracted out all Class II non-autonomous TEs with a length of 50–800 *nt* from Repbase as our reference dataset for comparison. In *Oryza sativa*, there are 217 Class II non-autonomous TEs annotated in Repbase, and 162 of them have a length of 50–800 *nt*. We used blastn (E-value ≤10^−10^) to compare the outputs of *MITE-Hunter*, *MITE Digger*, *RSPB*, and *detectMITE* with these 162 Repbase reference sequences. The comparison results are shown in Fig. 3 (the relevant data is available at: http://sourceforge.net/projects/detectmite/files/Supplementary_Data.7z).

As shown in Fig. 3, among 162 Repbase reference sequences, 48, 94, 15, and 49 are not detected by *MITE-Hunter*, *MITE Digger*, *RSPB*, and *detectMITE* respectively. Obviously, *RSPB* detected many more sequences in the Repbase data than other three tools. The major reason for this is that the *RSPB* detection result contains many sequences having short/diverse TIRs (*i.e.*, TIR pairs with a lower degree of pairing), low full-length copy number, or no flanking TSD, which will be compared and discussed in detail in the next section.

For the 49 sequences missed by *detectMITE*, we manually checked their structures and retrieved their full-length copies in genome using blastn. We found that 5 of them do not have complete TIR structures (ECSR, GLUTEL1LIKE, POP-OL2, TOURIST-XIII and WUJI), 27 have low full-length copy numbers that do not meet our cutoff of ≥3 (CASIN, COWARD-2, F1275, HEARTBLEEDING, ID-2, LIER, OSTE23, OSTE26, SEVERIN, STONE, TOURIST-XV, WUWU and STOWAWAY[15,16,19,24,25,26,27,28,29,30-2,30-3,31,35,40,42]_OS), 1 has high A/T content in its TIR (MUDRN4_OS), and 11 have too many mismatches (non-reverse complementary pairs) in the TIR (CASMALL, CASTAWAY-3, DITTO-2, DITTO3, EXPLORER, HELIA, ID-3, ID-4, NONAME, OSTE19 and THRIA). Among the 5 sequences that have full-length copies ≥3 (COWARD-3, DEBOAT, DELAY, STOWAWAY48_OS and TOUNJ-30), we further retrieved their flanking sequences, and found that only DELAY and STOWAWAY48_OS have at least 3 valid full-length copies bearing good TIRs and TSDs with different flanks. Therefore, *detectMITE* only missed 2 cases of the Repbase reference data.

The 48 Repbase reference sequences not detected by *MITE-Hunter* are CASIN, CASMALL, COWARD, COWARD-2, COWARD-3, DEBOAT, DITTO-2, DITTO3, ECSR, F1275, F770, FOCUS, GLUTEL1LIKE, HEARTBLEEDING, HELIA, ID-2, ID-3, ID-4, LIER, MUDRN4_OS, NONAME, OSTE23, OSTE24, OSTE26, POP-OL2, SEVERIN, STONE, STOWAWAY[15,16,19,21,24,25,26,27,30-2,30-3,30,31,32,40]_OS, TELIA, TOURIST-XI, TOURIST-XIII, TOURIST-XV, TOURIST6A_OS, WUJI and WUWU. Among them, COWARD, COWARD-3, DEBOAT, FOCUS, STOWAWAY21_OS and TOURIST6A_OS have full-length copies ≥3, and only COWARD, FOCUS and STOWAWAY21_OS have ≥3 valid full-length copies.

The 15 Repbase reference sequences missed by *RSPB* are CASIN, CASMALL, ECSR, F1275, HEARTBLEEDING, ID-2, LIER, OSTE23, OSTE26, OSTE28, POP-OL2, STOWAWAY19_OS, STOWAWAY40_OS, TOURIST-XIII and TWIF. Among them, only OSTE28 has more than 3 valid full-length copies.

Clearly, almost all MITEs in *Repbase* can be detected by *detectMITE*, *MITE-Hunter*, and *RSPB* effectively, while *MITE Digger* missed too many cases (*i.e.*, 94). In other words, the performance of *detectMITE*, *MITE-Hunter*, and *RSPB* in terms of Repbase annotation appears to be comparable. Although *RSPB* can match more sequences in the Repbase data, many of its so-called “complete” sequences still lack the complete and canonical structure of MITEs and/or do not meet our criteria for being a valid MITE member (see below).

### Comparison of *detectMITE* with *MITE-Hunter*, *MITE Digger*, and *RSPB* individually

Since *MITE-Hunter*, *MITE Digger*, and *RSPB* are the most popular tools for genome-wide detection of both known and novel MITEs, we compared MITE detection results in the rice genome between *detectMITE* and each of these three tools individually using blastn (E-value ≤10^−10^). For comparison purposes, the detection results have been divided into three categories: (1) sequences identified by both *detectMITE* and *MITE-Hunter* (or *MITE Digger*, *RSPB*), (2) sequences identified only by *MITE-Hunter* (or *MITE Digger*, *RSPB*), and (3) sequences identified only by *detectMITE*. All the relevant data for comparisons are available at http://sourceforge.net/projects/detectmite/files/Supplementary_Data.7z.

As described previously, different tools use different criteria to cluster similar MITE sequences into distinctive MITE families. In order to make the comparisons more convincing, we conducted all-against-all blastn for all representative sequences of 4790 MITE families detected by *detectMITE* and adopted the 80-80-80 rule utilized by *MITE-Hunter*^21^ to further cluster these MITE families into super-families. Accordingly, the aforementioned 4790 MITE families were classified into 1821 super-families.

**Figure 4:**
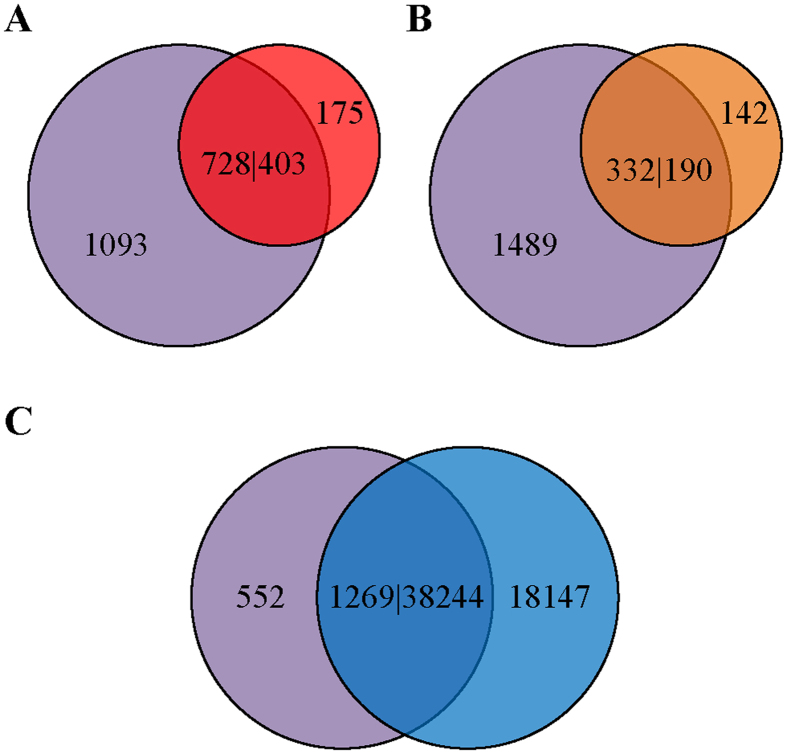
Comparison of MITEs detected in rice genome using *detectMITE* versus *MITE-Hunter*, *MITE Digger* and *RSPB* respectively. (**A**) Comparison between *detectMITE* and *MITE-Hunter*. **(B)** Comparison between *detectMITE* and *MITE Digger*. (**C**) Comparison between *detectMITE* and *RSPB*. The violet, red, brown, blue circles represent the outputs of *detectMITE*, *MITE-Hunter*, *MITE Digger* and *RSPB* respectively. The overlapping parts represent numbers of MITE sequences that match each other by blastn (E-value ≤10^−10^). Using (**A**) as an example, 728|403 means 728 MITE super-families in *detectMITE* output match 403 sequences in *MITE-Hunter* output, 1093 represents the number of MITE super-families in *detectMITE* output that do not match any sequences in *MITE-Hunter* output, and 175 represents the number of MITE sequences in *MITE-Hunter* output that do not match any super-families in *detectMITE* output.

As shown in Fig. 4A, 728 (or 728/1821 ≈ 40%) super-families (*i.e.*, 3397 MITE families) in *detectMITE* output match with 403 (or 403/578 ≈ 70%) consensus sequences in *MITE-Hunter* output, while 175 (or 175/578 ≈ 30%) consensus sequences in *MITE-Hunter* do not match any sequence in *detectMITE* output and 1093 (or 1093/1821 ≈ 60%) super-families (*i.e.*, 1393 MITE families) in *detectMITE* output do not match any sequence in *MITE-Hunter* output. In detailed analysis of these 175 sequences, we found that 76 of them have low full-length copy numbers (<3), 37 have high mismatch pairs in their TIRs, 23 do not bear a TIR, 1 has high A/T content in its TIR, and only 38 have full-length copies ≥3. Among these 38 cases, 21 have at least 3 valid full-length copies that bear good TIRs and TSDs and have distinct flanks. Therefore, this suggests that *detectMITE* missed 21 cases in comparison with *MITE-Hunter*, whereas *MITE-Hunter* missed 1093 super-families (*i.e.*, 1393 MITE families) identified by *detectMITE*.

In Fig. 4B, 332 (or 332/1821 ≈ 18%) super-families (*i.e.*, 2454 MITE families) in *detectMITE* output match 190 (or 190/332 ≈ 57%) sequences in *MITE Digger* output, whereas 142 (or 142/332 ≈ 43%) sequences in *MITE Digger* output do not match any sequence in *detectMITE* output and 1489 (or 1489/1821 ≈ 82%) super-families (*i.e.*, 2336 MITE families) in *detectMITE* output do not match any sequence in *MITE Digger* output. Among these 142 cases missed by *detectMITE*, 102 have low full-length copy numbers (<3), 10 have too many mismatch pairs in TIRs, 13 have high A/T (or G/C) content in TIRs, 3 have low similarity copies (similarity <80%), and 14 have full-length copies ≥3. We further checked the 14 cases with over 3 full length copies in the genome, and found that 4 of them have at least 3 valid full-length copies that possess canonical TIRs and TSDs with distinct flanks. Therefore, *detectMITE* only missed 4 cases in comparison with *MITE Digger*, where *MITE Digger* missed 1489 super-families (*i.e.*, 2336 MITE families) detected by *detectMITE*.

In Fig. 4C, 1269 (or 1269/1821 ≈ 70%) super-families (*i.e.*, 4021 MITE families) in *detectMITE* output match the 38244 (or 38244/56391 ≈ 68%) MITE sequences in *RSPB* output, whereas 18147 (or 18147/56391 ≈ 32%) sequences in *RSPB* output do not match any sequence in *detectMITE* output and 552 (or 552/1821 ≈ 30%) super-families (*i.e.*, 769 MITE families) in *detectMITE* output do not match any sequence in *RSPB* output. For 18147 sequences unique in *RSPB* output, we will check if they have more than 3 complete copies in the genome. Through clustering similar sequences by our criteria (*i.e.*, similarity ≥80% and coverage rate ≥99%), we obtained 13397 groups. Among them, only 795 groups have full-length copy number ≥3. Here, we do not consider the low copy number groups. For those groups that have copy number ≥3, we generated a multiple sequence alignment for each group and manually checked the alignment quality. Generally, they can be classified into the following categories (see Supplementary Fig. S2): (1) 46 groups do not have clear TIRs, (2) 344 groups contain too many mismatches in TIRs, (3) TIRs of 4 groups have high A/T content, (4) 305 groups have a low number of full-length copies with complete TIRs, and (5) 96 groups have complete TIRs with at least three full-length copies in the genome. For these 96 groups, we further retrieved their flanking sequences in genome and found that only 16 of them have ≥3 valid copies with a clear TIR and TSD and distinctive flanks. Therefore, *detectMITE* only missed 16 cases detected by *RSPB*, which possess canonical MITE structures and have at least 3 valid full length copies with distinctive flanks in the genome. On the other hand, 552 super-families (*i.e.*, 769 MITE families) reported by *detectMITE* are completely missed by *RSPB*.

For the MITE families uniquely detected by *detectMITE* in individual pair-wise comparisons with *MITE-Hunter*, *MITE Digger*, and *RSPB* respectively, we generated multiple sequence alignment for each family and manually examined the alignment results using BioEdit^48^. We found that all of these families meet our definition of canonical MITEs, having full-length copies ≥3, bearing clear TIR, and flanked by TSD (see Supplementary Fig. S3). More importantly, all the sequences identified by *detectMITE* are flanked by TSD, while many sequences in the output of *RSPB* do not meet this requirement.

Clearly, *detectMITE* misses some MITEs in detection in comparison with the aforementioned three tools, but it can detect many more MITEs than *MITE-Hunter* (1093 super-families/1393 families vs. 21), *MITE Digger* (1489 super-families/2336 families vs. 4), and *RSPB* (552 super-families/769 families vs. 16) in the rice genome. There are 509 super-families/669 families detected by *detectMITE* but missed by all three other tools (*MITE Digger*, *MITE-Hunter*, and *RSPB*). Moreover, when we adopted a looser clustering rule - the E-value of ≤10^−10^ rule used in *RSPB* (*i.e.*, two sequences will be classified into the same family/group if they have a valid blastn hit with an E-value of ≤10^−10^), the 4790 MITE families detected by *detectMITE* can be further clustered into 843 groups. Among these groups, 581, 703 and 335 do not have a valid match (E-value of ≤10^−10^) with the outputs of *MITE-Hunter*, *MITE Digger* and *RSPB* respectively. (http://sourceforge.net/projects/detectmite/files/Supplementary_Data.7z). Therefore, even with these two different loose clustering rules (*i.e.*, the 80-80-80 rule and the E-value of ≤10^−10^ rule), *detectMITE* still shows its capability of detecting many more MITEs than *MITE-Hunter*, *MITE Digger* and *RSPB*.

Moreover, the *detectMITE* output definitely contains fewer false positive cases of MITEs due to the structural requirement (*i.e.*, clear TIRs flanked by TSD) and copy number constraint (*i.e.*, full-length copy number of distinctive valid members with different flanking sequences ≥3) that we have enforced in our algorithms. If we examine the MITEs uniquely identified by *MITE-Hunter*, *MITE Digger* and *RSPB*, respectively, in comparison with *detectMITE*, these tools find many false positive MITEs that lack these important copy number and structural requirements. Among 175 consensus sequences reported by *MITE-Hunter* that do not match any sequence in the *detectMITE* detection output, 154 are false positives because only 21 have at least 3 valid full-length copies that possess good TIRs and TSDs with distinct flanks. Among 142 sequences detected by *MITE Digger* but not by *detectMITE*, 138 are false positives because only 4 have at least 3 valid full-length copies. Among 18147 sequences (or 795 groups) uniquely identified by *RSPB* but not by *detectMITE*, most of them appear to be false positives because only 16 groups have at least 3 valid full-length copies.

Since *MITE Digger* missed many more MITEs than *MITE-Hunter* and *detectMITE* in the rice genome, we extracted all 424 super-families (*i.e.*, 1003 MITE families) detected by *detectMITE* (http://sourceforge.net/projects/detectmite/files/Supplementary_Data.7z), which are shared by the outputs of *detectMITE* and *MITE-Hunter* but missed by *MITE Digger*, and blasted their representative sequences against the TIGR Plant Repeat Database^49^. The TIGR Plant Repeat Database contains various types of repeats sequences (including MITEs) in 12 plant genera (including rice). Among 424 super-families (*i.e.*, 1003 MITE families), 114 super-families (*i.e.*, 284 MITE families) matched the entries in TIGR Plant Repeat Database with the E-value cutoff of ≤10^−10^. Examples of such blast hits are shown in Supplementary Fig. S4. Furthermore, we also extracted all 1065 super-families (*i.e.*, 1333 MITE families) uniquely detected by *detectMITE* but missed by both *MITE Digger* and *MITE-Hunter*, and blasted their representative sequences against the TIGR Plant Repeat Database. Among them, 187 super-families (*i.e.*, 226 MITE families) matched the entries in the TIGR Plant Repeat Database with the E-value cutoff of 10^−10^. Examples of such blast hits are show in Supplementary Fig. S5. Since the repeat sequences in TIGR Plant Repeat Database were obtained using homology-based methods that take advantages of GenBank and other public annotations^49^, the likelihood that these matched MITEs are real MITEs is high. Clearly, these results can demonstrate the reliability of *detectMITE* in finding novel MITEs.

## Discussion

To fully elucidate the origins, functions, and biological relevance of MITEs, we need to comprehensively, accurately, and effectively detect the ubiquitous MITEs hidden in eukaryotic genomes. Due to the well-defined structures of MITEs, many tools are available for performing MITE detection. However, the complex organizations and compositions of genomes make the accurate, comprehensive, and effective detection of MITE very challenging. That explains why accurate and effective tools for MITE detection are currently rare. *FINDMITE* and *MUST* are structure-based methods for MITE detection, but have high false-positive rates in their outputs and cannot deal with genome-scale inputs^21^. Homology-based methods can only detect known MITEs and are mostly applicable in the discovery of MITEs between closely related genomes^22^. Using both *de novo* and structure-based approaches, *MITE-Hunter* and *MITE Digger* clearly improve the accuracy of genome-wide MITE detection, but can only detect a portion of MITEs hidden in genomes^21^,^28^,^29^. *RSPB* is essentially a mixture of both *de novo* and homology-based methods, but generates outputs that often include lots of sequences without a typical or complete structure of canonical MITEs. Furthermore, *RSPB* is time- and resource-consuming in its execution.

From our data analysis using the rice genome, it is clear that *detectMITE* can more comprehensively and accurately detect MITEs than the three popular tools for MITE detection. *detectMITE* is faster than *MITE Digger*, which is considered the most efficient tool in MITE detection so far^29^. As mentioned previously, *detectMITE* cannot detect MITEs that bear indels in their terminal inverted repeats. Nevertheless, the numerical approach for searching inverted repeats, either perfect ones or imperfect ones with mismatched/non-complementary pairs, can be more exhaustive and comprehensive than conventional string matching approaches^35^,^36^. This is why *detectMITE* is capable of detecting many more MITEs with a complete and canonical MITE structure hidden in genomes than popular string matching tools, even with its inability to detect MITEs with indels within TIRs. *detectMITE* has taken advantage of robust vector calculation power of MATLAB, which explains why *detectMITE* is very efficient in its detection.

Using the Lempel-Ziv complexity algorithm, *detectMITE* can identify many low complexity sequences that *MITE-Hunter* and *RSPB* cannot find. *detectMITE* adopted the notion that sequence similarities are only shared in the internal sequences of different members in a MITE family, whereas the flanking sequences are not supposed to be transposed together^21^,^28^. Then, *detectMITE* uses a PSA (Pairwise Sequence Alignment) method to find the number of valid full-length members (copies) in a given family that bear different flanking sequences^21^. Clustering of similar MITE sequences into distinctive MITE families is the most time-consuming and resource-demanding process in MITE detection. *detectMITE* utilizes the more efficient clustering program *CD-HIT* to replace blastn and ensures that only highly similar sequences (≥80%) with high coverage (≥99%) can be clustered together.

As the rice genome is the well-studied genome in MITEs research, we used the rice genome as our test data to evaluate the performance and reliability of *detectMITE* in MITE detection. In comparison with known MITEs annotated in Repbase, *detectMITE* missed 2 cases, *MITE-Hunter* missed 3 cases, and *RSPB* missed 1 case, demonstrating that *detectMITE*, *MITE-Hunter*, and *RSPB* have comparable abilities in annotating known MITEs accurately. Compared to *MITE-Hunter*, *MITE Digger* and *RSPB*, *detectMITE* performs with higher efficiency and can detect many MITEs that are missed by these tools, as well as by Repbase (see Figs 3 and 4). Although *detectMITE* certainly misses some cases when compared with these tools, it can detect many more sequences that meet the criteria of MITEs than *MITE-Hunter* (1093 super-families vs. 21), *MITE Digger* (1489 super-families vs. 4), and *RSPB* (552 super-families vs. 16). Even with loose clustering criteria (*i.e.*, *RSPB*’s E-value of ≤10^−10^ rule), *detectMITE* still demonstrates its advantage of finding more MITEs than its competitors. More importantly, the detection result of *detectMITE* clearly contains fewer false positives due to the structure constraint (*e.g.*, with clear TIR and TSD) and copy number constraint (at least 3 valid, full-length copies with different flank sequences). This makes *detectMITE* competitive in MITE detection, since detection results of *MITE-Hunter*, *MITE Digger* and *RSPB* often contain many false positives, requiring tedious manual checks. Furthermore, *detectMITE* provides information on accurate positions and length of flanking TSDs for each sequence in its output.

In conclusion, we present a novel numeric-calculation-based program *detectMITE* that can more comprehensively, accurately, and effectively identify MITEs in genomes than other available tools. Without a doubt, *detectMITE* is a valuable addition to the research community studying MITEs and other transposon elements. Computational methods, however, can only utilize different features of MITEs (*e.g.* sequence structures and similarities, as well as genome-wide copy numbers) to justify whether a candidate sequence is a valid MITE or not. To determine whether a novel candidate is a genuine MITE or not in reality, further wet-lab experiments are clearly needed. In the future, we will work to improve the core algorithm so that terminal inverted repeats with indels in the paring stem can be detected using numeric calculation approaches. Also, a mixed strategy that integrates homology-based approaches, *e.g.*, blast search for well-defined MITE families detected by *detectMITE*, can be used to annotate additional potential MITEs in genomes.

## Additional Information

**How to cite this article**: Ye, C. *et al.* *detectMITE*: A novel approach to detect miniature inverted repeat transposable elements in genomes. *Sci. Rep.* **6**, 19688; doi: 10.1038/srep19688 (2016).

## Supplementary Material

Supplementary Information

## Figures and Tables

**Table 1:** The numbers of MITEs detected in the rice genome using *detectMITE*, *MITE-Hunter*, *MITE Digger* and *RSPB* respectively.

Program	Processing Time	Number of MITE Sequences	Number of MITE Families
*detectMITE*	10.79 hrs*	35,969	4,790
*MITE-Hunter*	28.01 hrs*	/	631
*MITE Digger*^a^	15.44 hrs	/	332
*RSPB*^b^	/	179,415	497

^*^All tests were conducted using an Ubuntu 12.04 (precise) 64-bit computer with Intel Xeon (2.00 GHz) processors, 4 CPU cores, and 128 GB RAM.

^a^The result is obtained from the publication of *MITE Digger*^28^.

^b^The result is obtained from P-MITE database^29^. Among 179415 MITEs reported by *PSPB*, only 56391 (*i.e.*, 31.4%) were labeled as complete sequences that were supposed to have complete terminal inverted repeats, whereas the others were labeled as partial sequences^29^.
