# Supplementary material for: detectMITE: A novel approach to detect miniature inverted repeat transposable elements in genomes
Source: Sci Rep. 2016 Jan 22;6:19688. doi: 10.1038/srep19688 (PMC4726161; doi:10.1038/srep19688)
Supplement: Supplementary Information [file srep19688-s1.doc]

***Supplementary Information***

***for***

***detectMITE*: A novel approach to detect miniature inverted repeat**

**transposable elements in genomes**

Congting Ye, Guoli Ji and Chun Liang

**Supplementary Figures**

Figure S1 …………………………………………………………………………… 2

Figure S2 …………………………………………………………………………… 3

Figure S3 …………………………………………………………………………… 4

Figure S4 …………………………………………………………………………… 5

Figure S5 …………………………………………………………………………… 6

**Figure S1. Examples of low complexity MITEs identified by the Lempel-Ziv complexity algorithm in the rice genome from the detection outputs of *MITE-Hunter* and *RSPB*.**

(**A**) A sequence containing tandem repeats in the output of *RSPB*. (**B**) A sequence mainly consisting of ‘AT’ dinucleotide repeats in the output of *RSPB*. (**C**) A sequence containing too many unknown bases in the output of *RSPB*. (**D**) A sequence containing tandem repeats in the output of *MITE-Hunter*.

**Figure S2.** **Examples of the 795 groups of MITE sequences in the rice genome uniquely identified by *RSPB*, but not by *detectMITE*.**

**(A)** Sequences that do not bear terminal inverted repeats (TIRs). **(B)** TIRs of sequences that have too many mismatches or non-complementary pairs. **(C)** A/T content of TIRs is too high. **(D)** Number of full-length copies of the MITE sequences possessing good TIRs is less than 3 across the genome (*i.e.*, the second and the last sequences do not have good TIRs - mismatched pairs in the stem≥3). Relevant data are available at [http://sourceforge.net/projects/detectmite/files/Supplementary_Data.7z](http://sourceforge.net/projects/detectmite/files/Supplementary_Data.7z/).


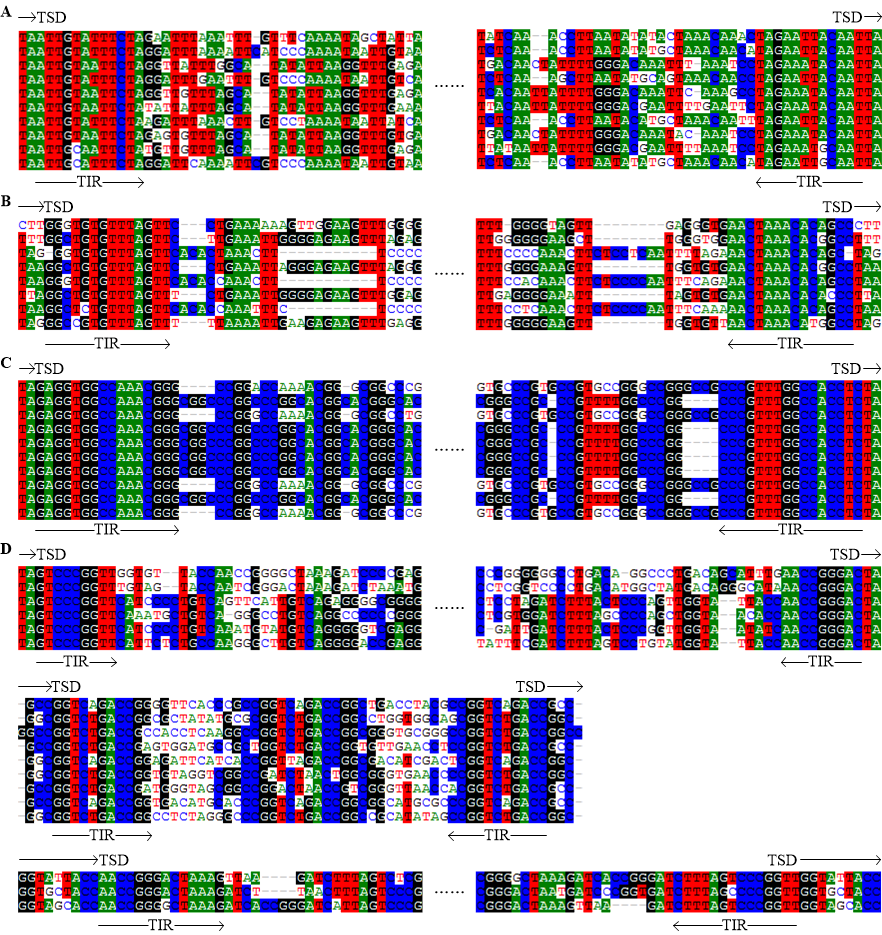


**Figure S3. Examples of MITE super-families (represented by family member) uniquely detected by *detectMITE* among the pairwise comparison with *MITE Digger*, *MITE-Hunte***r**, and *RSPB* individually.**

(**A**) A MITE super-family (family_2578 in super-family_1295) missed by *MITE Digger*. (**B**) A MITE super-family (family_3151 in super-family_1445) missed by *MITE-Hunter*. (**C**) A MITE super-family (family_4493 in super-family_1757) missed by *RSPB*. (**D**) Three MITE super-families (family_3080 in super-famiy_1419; family_1254 in super-family_660; family_1151 in super-family_587) missed by *MITE Digger*, *MITE-Hunter* and *RSPB* together. Relevant data are available at <http://sourceforge.net/projects/detectmite/files/Supplementary_Data.7z>.

**Figure S4. Examples of MITE super-families (represented by family member) detected by *detectMITE*, shared by *MITE-Hunter* but missed by *MITE Digger*, have valid blast matches (e-value** ≤**10^-10^) against the TIGR Plant Repeat Database.**

In each alignment, the upper sequence is the MITE sequence detected by *detectMITE*, and the lower sequence is the sequence annotated in the TIGR Plant Repeat Database. | stands for two identical nucleotides. : stands for two different nucleotides. - stands for a gap. Relevant data are available at <http://sourceforge.net/projects/detectmite/files/Supplementary_Data.7z>.

**Figure S5. Examples of MITE super-families (represented by family member) detected by *detectMITE* but missed by both *MITE Digger* and *MITE-Hunter* have valid blast matches (e-value** ≤**10^-10^) against the TIGR Plant Repeat Database.**

In each alignment, the upper sequence is the MITE sequence detected by *detectMITE*, and the lower sequence is the sequence annotated in the TIGR Plant Repeat Database. | stands for two identical nucleotides. : stands for two different nucleotides. - stands for a gap. Relevant data are available at <http://sourceforge.net/projects/detectmite/files/Supplementary_Data.7z>.
